# The complete chloroplast genome of *Epimedium mikinorii* Stearn. (Berberidaceae)

**DOI:** 10.1080/23802359.2020.1715863

**Published:** 2020-01-24

**Authors:** Chunmei Wen, Xiang Liu, Yu Yao, Yanjiao Luo, Ting Liu, Qianru Yang, Baolin Guo, Chaoqun Xu, Fengmei Suo, Guoan Shen, Fei Ge

**Affiliations:** a School of Pharmacy, Jiangxi University of Traditional Chinese Medicine, Nanchang, China;; b Institute of Medicinal Plant Development, Chinese Academy of Medical Science, Peking Union Medical College, Beijing, China;; c Chongqing Academy of Chinese Materia Medica, Chongqing, China;; d School of Pharmacy, Shanxi Medical University, Taiyuan, China;; e College of Traditional Chinese Medicine, Shanxi University of Chinese Medicine, Jinzhong, China

**Keywords:** Chloroplast genome, *Epimedium mikinorii*, Berberidaceae

## Abstract

*Epimedium mikinorii* is a vulnerable species in the *Epimedium* genus of Berberaceae. Here, we sequenced the complete chloroplast genome of *E. mikinorii*, which is 157,136 bp in length, and is a typical quadripartite circular molecule composed of two inverted repeats (IRs) of 25,896 bp for each, a large single-copy region (LSC) of 88,395 bp, and a small single-copy region (SSC) of 16,949 bp. The complete chloroplast genome of *E. mikinorii* contains 134 genes, including 83 protein-coding genes, 38 tRNA genes, 8 rRNA genes, and 5 pseudogenes. Phylogenetic analysis showed that *E. mikinorii* was closely related to *E. dolichostemon*.

*Epimedium* is famous for its medicinal and ornamental value. Most of the species in this genus are distributed mainly in the southwest and central regions of China (Zhang et al. 2014; Li et al. 2016). Epimedii folium has the therapeutic effects of nourishing kidney, strengthening bones and relieving rheumatism, and has a long history of medical use in traditional Chinese medicine (TCM) (Ye and Chen 2001; Wu et al. 2003; Ma et al. 2011). The taxonomy and phylogeny of the genus *Epimedium* is controversial. It is well known that the chloroplast genome can provide effective information for studying the phylogenetic relationship of species (Nguyen et al. 2015). So far, the complete chloroplast genomes of six species of *Epimedium* have been reported (Liu et al. 2019; Zhang et al. 2016). In this study, we sequenced the complete chloroplast genomes of a vulnerable species of *E. mikinorii*, aiming to provide valuable information for studying taxonomy and phylogeny of *Epimedium* genus.

The *E. mikinorii* samples in this study were collected in the Enshi City, Hubei Province (China; N31°19′, E109°23′), and a voucher specimen (0431) was deposited in the Herbarium of the Institute of Medicinal Plant (IMPLAD), Beijing, China. Total DNA was extracted from the fresh leaves of *E. mikinorii* by modified CTBA (Doyle and Doyle 1987). Sequencing was carried out on the Illumina Novaseq PE150 platform, and the pair-end reads of 150 bp was generated. The filtered reads were assembled into a complete chloroplast genome by the program GetOrganelle v1.5 (Jin et al. 2018), using the *E. acuminatum* chloroplast genome (GenBank accession number: NC_029941) as a reference. The annotation of the complete chloroplast genome was carried out through the online annotators of both CPGAVAS2 (Shi et al. 2019) and Geseq (Tillich et al. 2017), followed by manual correction. In order to explore the phylogenetic relationship of *E. mikinorii*, we downloaded the whole chloroplast genomes of 19 plant species from the NCBI GenBank database. Maximum likelihood (ML) analyses were performed using raxmlGUI 1.5 b (v8.2.10) (Silvestro and Michalak 2012).

The chloroplast genome of *E. mikinorii* (GenBank accession number: MN857416) is 157,136 bp in length, which is a typical quadripartite circle composed of a pair of reverse repeating region (IRa and IRb, 25,896 bp), a large single-copy region (LSC, 88,395 bp), and a small single-copy region (SSC, 16,949 bp). The chloroplast genome contains 134 genes, including 83 protein-coding genes, 38 tRNA genes8 rRNA genes, and 5 pseudogenes (*ψinfA, ψrpl2, ψycf15 × 2,* and *ψycf1*). Most of these genes are single copy (15 of them have one intron, three genes of *ycf3, clpP,* and *rps12* contain two introns), whereas five protein-coding genes (*ycf2, ndhB, rps7, rpl23,* and *rps12*), seven tRNA genes (*trnL-CAA, trnI-GAU, trnV-GAC, trnA-UGC, trnR-ACG, trnN-GUU,* and *trnI-CAU),* four rRNAs genes *(rrn4.5, rrn5, rrn16,* and *rrn23*), and one pseudogenes (*ψycf15*) are replicated in the IR region, and one tRNA gene (*trnQ-UUG*) is duplicated in the large single-copy region (LSC). The total content of GC is 38.81% in *E. mikinorii* chloroplast genome, while the content of GC in IR, LSC, and SSC regions is 43.15%, 37.42%, and 32.72%, respectively.

The phylogenetic analyses showed that *E. mikinorii* and *E. dolichostemon* were clustered together, indicating that the evolutionary relationship between them is closer (Figure 1). The determination of the chloroplast genome of *E. mikinorii* provides valuable information regarding the taxonomy and phylogeny of Berberaceae. 

**Figure 1.**
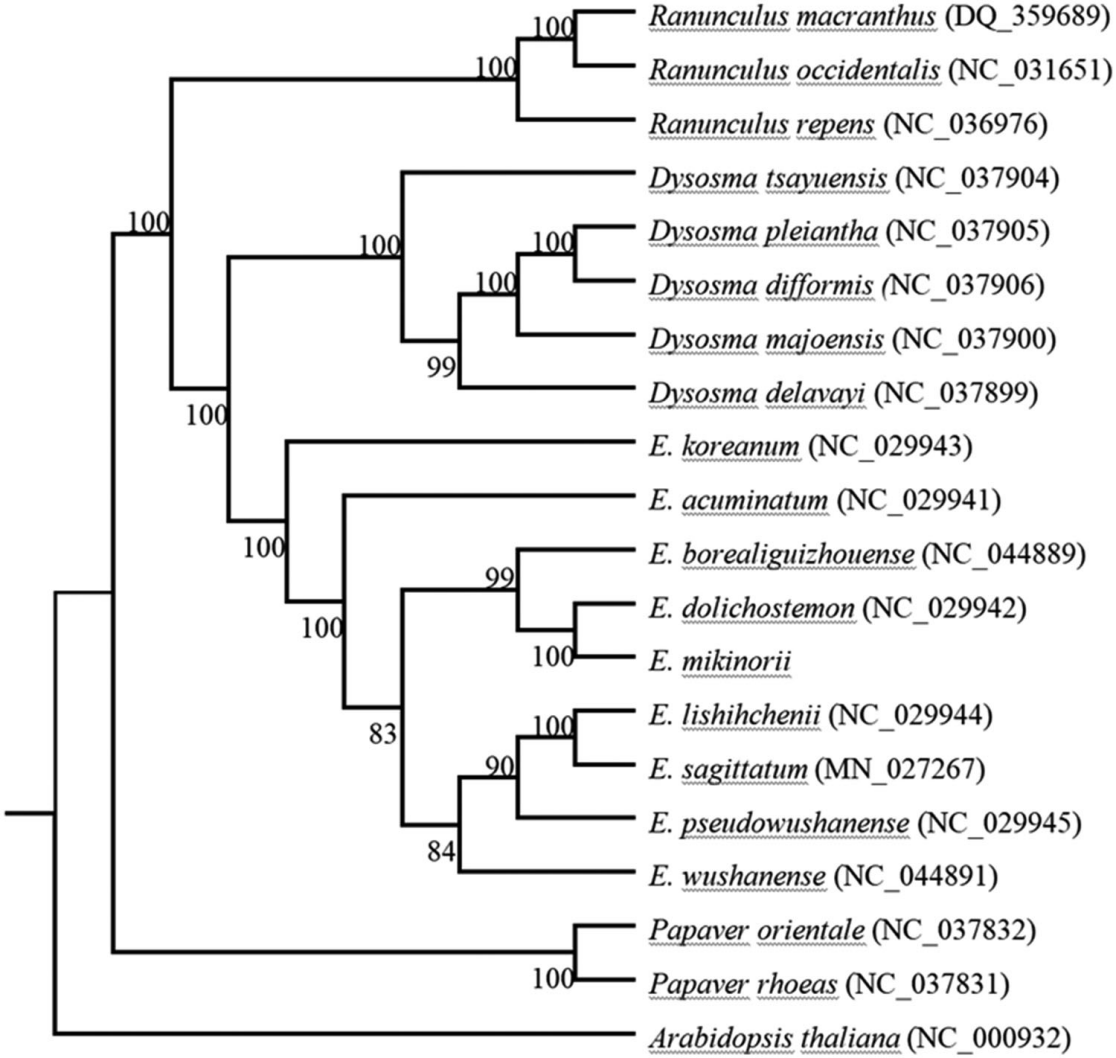
Phylogenetic analysis of *E. mikinorii* with other plant species. The phylogenetic tree was constructed based on the whole chloroplast genome sequences from 20 species in Ranales with Maximum likelihood (ML), with *Arabidopsis thaliana* as an outgroup. Numbers above node are bootstrap support values (>50%).
